# Smart city development and medical service performance: evidence from a province-level panel study in China

**DOI:** 10.3389/ijph.2026.1609627

**Published:** 2026-05-04

**Authors:** Xu Shao, Xi Liu, Jie Che, Bing Yang

**Affiliations:** 1 School of Law and Public Administration, Leshan Normal University, Leshan, China; 2 Department of Economics and Trade, Sichuan Business Vocational College, Chengdu, China; 3 Department of Tourism Management, Sichuan Business Vocational College, Chengdu, China

**Keywords:** China, digital governance, healthcare systems, medical service performance, smart city development

## Abstract

**Objectives:**

This study examines whether smart city development is associated with improvements in medical service performance across Chinese provinces, addressing the limited large-scale quantitative evidence on digital governance and healthcare systems in developing-country contexts.

**Methods:**

A balanced province-level panel dataset covering 31 provinces in mainland China from 2013 to 2024 was constructed using national statistical yearbooks and official smart city pilot lists. Medical service performance was measured by a composite Medical Service Performance Index (MSPI) capturing healthcare utilization structure, care quality, and preventive health management capacity. Two-way fixed effects models were applied to estimate the association between smart city exposure and healthcare system performance.

**Results:**

Higher smart city exposure is associated with improved medical service performance. In baseline models, smart city exposure is positively related to MSPI (β = 0.182, p < 0.01). Mechanism analyses show improvements in preventive health management (β = 0.184, p < 0.01), care quality (β = 0.135, p < 0.01), and reduced reliance on high-intensity hospital services (β = −0.120, p < 0.01). After accounting for these mechanisms, the direct association becomes statistically insignificant.

**Conclusion:**

Smart city development is linked to better medical service performance in China, suggesting that digital governance initiatives may strengthen healthcare systems through improved coordination, care quality, and preventive health management.

## Introduction

The rapid diffusion of smart city initiatives has reshaped the ways in which urban systems deliver public services, including healthcare [1]. By embedding digital technologies into urban governance, smart cities aim to enhance service accessibility, efficiency, and quality, while addressing persistent challenges such as resource constraints, fragmented service provision, and rising health demands. In the healthcare sector, smart city development has increasingly been viewed as a potential tool for strengthening health systems and improving medical service performance [2].

From a functional perspective, digital technologies integrated into smart city infrastructures may influence healthcare systems through several pathways. First, digital platforms such as telemedicine, mobile health applications, and remote monitoring technologies can reduce spatial and informational barriers, thereby improving access to healthcare services [3–5]. Second, smart city applications may contribute to better clinical outcomes by enabling earlier diagnosis, continuous health monitoring, and improved coordination among healthcare providers [6–8]. Third, digital governance tools can enhance the efficiency of healthcare systems by optimizing resource allocation, reducing waiting times, and improving service coordination across institutions [9]. Together, these mechanisms suggest that smart city initiatives may affect healthcare systems not only through technological innovation but also through broader transformations in urban governance and public service delivery.

Despite these potential benefits, empirical evidence on the relationship between smart city development and healthcare system performance remains limited. Much of the existing research relies on qualitative case studies or conceptual discussions focusing on individual technologies or pilot projects [10, 11]. As a result, relatively little large-scale quantitative evidence exists on whether smart city development leads to measurable improvements in healthcare system performance. Moreover, existing studies rarely examine how the effects of digital governance initiatives vary across regions with different levels of economic development, healthcare infrastructure, and institutional capacity. These questions are particularly relevant in developing countries, where regional disparities in resources and governance capacity remain substantial.

China provides a valuable context for examining these issues. Over the past decade, the Chinese government has actively promoted smart city development through a series of national pilot programs, leading to the widespread diffusion of digital governance initiatives across cities and regions [12]. At the same time, China’s healthcare system exhibits significant regional variation in both resource availability and service capacity. This combination of policy diffusion and regional heterogeneity provides a useful setting for assessing how smart city development may influence healthcare system performance.

Against this background, this study examines the relationship between smart city development and medical service performance in China. Using province-level panel data and a two-way fixed effects framework with continuous treatment intensity, the analysis evaluates whether greater exposure to smart city initiatives is associated with improvements in a composite Medical Service Performance Index. This study contributes to the literature in three ways. First, it extends existing research on digital transformation and healthcare by examining smart city development as a broader form of urban digital governance rather than focusing solely on individual medical technologies. Second, it provides large-scale empirical evidence on the relationship between smart city development and healthcare system performance using province-level panel data. Third, by examining changes in healthcare utilization structure, clinical service quality, and preventive health management, the study sheds light on the mechanisms through which digital governance initiatives may influence healthcare systems.

### Smart cities as digital governance

The understanding of smart cities has gradually shifted from a technology-driven perspective toward a broader governance-oriented framework. Earlier studies largely focused on digital infrastructures—such as the Internet of Things (IoT), cloud computing, and artificial intelligence—as the primary forces behind improvements in urban services [13]. Within this view, technological deployment itself was expected to generate efficiency gains and service improvements [14].

Recent research, however, increasingly interprets smart cities as a transformation of governance arrangements rather than a simple expansion of technological capacity [15]. Digital infrastructures serve primarily as enabling platforms that reshape information flows, coordination among institutions, and decision-making processes within urban systems [16]. As a result, the effectiveness of smart city initiatives depends not only on the availability of advanced technologies but also on how these tools are incorporated into existing institutional settings and public service structures.

This perspective is particularly relevant in the healthcare sector. Healthcare systems often involve fragmented service provision, strong interdependence among institutions, and substantial information asymmetry between providers and patients. In such environments, isolated technological applications rarely lead to substantial improvements in overall system performance. The benefits of digital technologies are more likely to emerge when they support coordination across providers, facilitate information sharing, and improve the integration of health-related services. For this reason, smart city initiatives may affect healthcare performance not only through technological innovation but also through broader improvements in governance capacity and institutional coordination.

### A multidimensional perspective on healthcare system performance

Within health policy and public administration research, healthcare system performance is commonly viewed as a multidimensional concept. Long-standing debates have explored questions concerning the objectives of healthcare systems, the role of medical services in shaping health outcomes, and whether healthcare should primarily be regarded as a public good or as a market-based service [17]. In response to these debates, contemporary research increasingly evaluates healthcare performance across several dimensions rather than relying on single indicators [18].

In line with this perspective, healthcare system performance in this study is examined through three interrelated dimensions: the structure of healthcare utilization, the quality of medical services, and the capacity for preventive health management.

The structure of healthcare utilization reflects the efficiency with which medical resources are used. Excessive reliance on high-intensity hospital-based services—such as avoidable hospitalizations or unnecessary admissions to tertiary hospitals—often indicates inefficiencies within healthcare systems. Improving healthcare performance therefore requires a more balanced pattern of service use, including greater reliance on community-level care and more appropriate substitution between different types of services [19].

The quality of healthcare services captures the effectiveness and safety of medical treatment. This dimension is often assessed through outcome-based indicators such as mortality rates or treatment success rates, which reflect the clinical performance of healthcare providers [20].

The third dimension concerns preventive health management, which emphasizes population-oriented services such as chronic disease management, maternal and child healthcare programs, and community-based health monitoring. These activities contribute to improved health outcomes by focusing on prevention, early detection, and long-term health maintenance rather than solely on treatment [21].

The concept of smart cities has evolved from a technology-centered paradigm toward a governance-oriented framework. Early perspectives emphasized digital infrastructures—such as IoT systems, cloud computing, and artificial intelligence—as primary drivers of service improvement [22].

Considering these dimensions together highlights the broader role of healthcare systems in maintaining population health. Improvements in healthcare performance typically occur through gradual adjustments in service organization, resource allocation, and health management practices rather than through isolated technological interventions.

### Smart cities and healthcare system performance

Smart city development may influence healthcare system performance through several channels related to information integration, service coordination, and health management.

One important pathway concerns changes in healthcare utilization patterns. Digital infrastructures, including online consultation platforms, digital appointment systems, and telemedicine services, can reduce unnecessary hospital visits while enabling patients to access appropriate care more efficiently. These technologies may encourage a shift away from hospital-centered treatment toward a more diversified system that includes remote services and community-level healthcare provision, thereby improving the efficiency of resource use within the healthcare system [23].

A second pathway relates to improvements in healthcare quality. Smart city platforms often integrate electronic health records, environmental monitoring systems, and large-scale urban data platforms. Access to more comprehensive and timely information can assist healthcare providers in clinical decision-making while also enabling greater coordination among medical institutions. Enhanced data sharing may reduce information asymmetries and support more consistent treatment practices across healthcare providers [24].

Smart city technologies may also strengthen preventive health management. Devices such as wearable health monitors, mobile health applications, and urban sensor networks enable continuous observation of health behaviors and environmental risks. These technologies support disease surveillance, early risk detection, and targeted health interventions. In many cities, digital platforms are also used to promote healthy lifestyles, support active aging programs, and expand community-based healthcare services, reinforcing the population-oriented functions of healthcare systems [25].

Although research on smart cities and digital health has expanded in recent years, important gaps remain. A substantial share of the literature focuses on specific technologies or individual city case studies, while systematic quantitative analyses examining the broader relationship between smart city development and healthcare system performance remain limited. Moreover, relatively few studies integrate smart city governance perspectives with multidimensional frameworks of healthcare system performance.

To address these gaps, this study uses province-level panel data from China and constructs a multidimensional Medical Service Performance Index (MSPI). The index captures three key aspects of healthcare systems: service utilization structure, healthcare quality, and preventive health management capacity. This approach allows a systematic evaluation of how smart city development relates to different aspects of healthcare system performance.

Based on the theoretical discussion above, the following hypotheses are proposed:

H1 Higher levels of smart city development are associated with improved medical service performance.

H2 Smart city development improves medical service performance by optimizing healthcare utilization structures and promoting service substitution.

H3 Smart city development improves medical service performance by enhancing healthcare quality and strengthening preventive health management capacity.

## Methods

### Data sources

This study uses a balanced province-level panel dataset covering all 31 provinces in mainland China from 2013 to 2024. The starting year of 2013 coincides with the launch of the national smart city pilot program, allowing the analysis to capture the diffusion of smart city initiatives across provinces over time. Using this extended time span also ensures sufficient temporal variation in the key explanatory variable for reliable econometric identification.

Information on medical service delivery and health management was obtained from the China Health Statistics Yearbook and the China Health and Family Planning Statistical Yearbook. Data on smart city development were collected from official lists of national smart city pilot programs released by central and local governments.

Socioeconomic and demographic control variables, including gross domestic product (GDP) *per capita*, urbanization rate, population density, and *per capita* fiscal expenditure, were drawn from the China Statistical Yearbook and related official statistical publications. All variables were aggregated at the provincial level. As the study relies exclusively on publicly available secondary data, ethical approval and informed consent were not required.

### Medical service performance

Healthcare system performance is measured using the Medical Service Performance Index (MSPI), a composite indicator capturing multiple aspects of medical service delivery and population health management at the provincial level. The index follows multidimensional evaluation frameworks commonly used in health policy and public administration research [26]. These frameworks emphasize that healthcare system performance reflects not only service volume but also resource allocation efficiency, service quality, and the provision of preventive health services [27].

Consistent with this perspective, MSPI incorporates three conceptual dimensions: healthcare utilization structure, healthcare quality, and preventive health management capacity. The utilization dimension reflects whether healthcare resources are allocated efficiently and whether unnecessary reliance on high-intensity hospital-based services can be reduced. The quality dimension captures clinical outcomes and service safety, consistent with the well-known structure–process–outcome framework in healthcare evaluation [28]. The preventive health management dimension reflects the capacity of healthcare systems to provide population-oriented services such as maternal healthcare, chronic disease management, and community health monitoring.

Seven indicators were selected to operationalize these dimensions: outpatient visits, hospital admissions, emergency mortality rate, inpatient mortality rate, coverage of standardized health management services, prenatal examination utilization, and newborn follow-up visits. These indicators capture both treatment-oriented and preventive aspects of healthcare provision and have been widely used in previous evaluations of health system performance [29].

To ensure comparability across indicators with different units and distributions, all variables were standardized using pooled z-scores (Equation 1). The standardized value of each indicator is calculated as

$$Z_{kpt}=\frac{X_{kpt}‐{\bar{X}}_{k}}{\sigma_{k}}$$ (1)

where *X*_*kpt*_ denotes the original value of indicator *k* in province *p* and year *t*; ${\bar{X}}_{k}$ represents the sample mean of indicator *k* calculated across all provinces and years; and $\sigma_{k}$ denotes the corresponding standard deviation.

Indicators reflecting adverse outcomes (e.g., mortality rates) were reverse-coded before standardization so that higher values consistently represent better performance. The MSPI is then calculated as the unweighted mean of the standardized indicators:

$$MSPI_{pt}=\frac{1}{K}\sum_{k=1}^{K}Z_{kpt}$$ (2)

where *Z*_*kpt*_ denotes the standardized value of indicator *k* in province *p* and year *t*, and KKK represents the total number of indicators included in the index (Equation 2). Equal weighting is commonly used in public health research when no clear theoretical or empirical basis exists for assigning differential weights across indicators [30].

For additional analysis, the standardized indicators are also aggregated into three sub-indices corresponding to the conceptual dimensions of healthcare utilization, healthcare quality, and preventive health management. These indices are used in the mechanism analysis to examine whether changes in overall healthcare performance are associated with improvements in specific dimensions of the healthcare system.

### Key explanatory variable

The key explanatory variable is smart city exposure, defined as the proportion of cities within a province designated as national smart city pilots in a given year.

China’s national smart city pilot program began in 2013 when the Ministry of Housing and Urban-Rural Development released the first list of pilot cities together with supporting policy guidelines. Since then, pilot programs have expanded gradually across different regions [31].

Smart city exposure is calculated as

$$Smart\;Exposure_{pt}=\frac{{\;N}_{pt}^{\;smart}}{N_{p}^{\;total}}$$ (3)

Where $N_{pt}^{smart}$ denotes the number of pilot cities in province p in year t, and $N_{p}^{total}$ denotes the total number of cities within province.

This measure reflects the spatial diffusion of smart city initiatives within provinces (Equation 3). The indicator implicitly assumes that each pilot city contributes equally to provincial exposure. In practice, however, cities differ in fiscal capacity, governance capability, and digital infrastructure conditions. The variable should therefore be interpreted as a measure of policy diffusion rather than a direct measure of implementation intensity. This limitation is discussed further in the Discussion section.

This operationalization implicitly assumes that each smart city pilot contributes equally to the provincial level of smart city exposure. However, in practice, cities differ substantially in fiscal capacity, governance capability, and digital infrastructure conditions, which may lead to variations in the intensity and effectiveness of policy implementation [32]. Therefore, the smart city exposure indicator used in this study should be interpreted primarily as a measure of the spatial diffusion of smart city policies rather than a precise measure of implementation quality. This measurement limitation is further discussed in the Discussion section.

**FIGURE 1:**
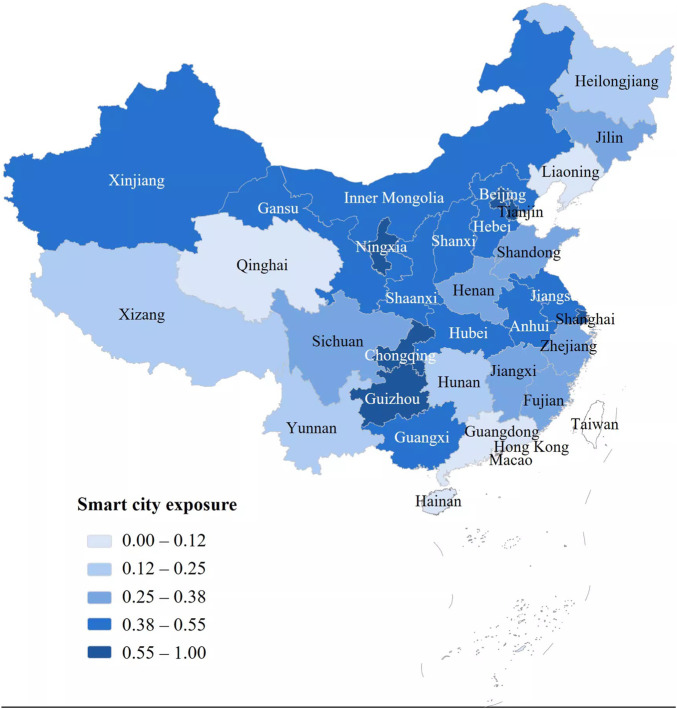
Spatial distribution of smart city exposure across provincial regions. Smart City Development and Medical Service Performance Study, China, 2013-2024.

Figure 1 illustrates the spatial distribution of smart city exposure across Chinese provinces. The map uses quantile classification to group provinces into five exposure levels. Considerable regional variation is observed. Provinces with larger urban systems tend to show higher exposure levels, while several western regions display relatively low exposure.

### Control variables

Several time-varying provincial characteristics are included to account for potential confounding factors. These variables represent key aspects of economic development, population structure, and fiscal capacity.

Economic development is measured by GDP *per capita* [33]. Urbanization rate reflects population concentration and demand for public services [34]. Population density captures differences in healthcare utilization pressure and the spatial distribution of medical resources. Fiscal capacity is measured by *per capita* fiscal expenditure, indicating local governments’ ability to finance healthcare services and support digital governance initiatives.

These variables are widely used in empirical studies examining healthcare system performance and public sector digitalization.

### Statistical analysis

The association between smart city exposure and healthcare system performance is estimated using a two-way fixed effects (TWFE) panel regression model:

$$MSPI_{pt}=\beta \;SmartExposure_{pt}+\gamma X_{pt}+\mu_{p}+\lambda_{t}+\varepsilon_{pt}$$ (4)

where *MSPI*_*pt*_ denotes medical service performance in province *p* and year *t*; *Smart Exposure*_*pt*_ represents smart city exposure and *X*_*pt*_ is a vector of time-varying control variables. Province fixed effects (*μ*_*p*_)account for time-invariant provincial characteristics, while year fixed effects(*λ*_*t*_)capture nationwide shocks affecting all provinces.

The model exploits within-province variation in smart city exposure over time (Equation 4). Standard errors are clustered at the provincial level to account for serial correlation.

### Mechanism and robustness analysis

Potential mechanisms are examined using three indices derived from the MSPI components: the service substitution index (SSI), the quality improvement index (QII), and the health management index (HMI). These indices correspond to the three conceptual dimensions of healthcare system performance.

The SSI captures changes in healthcare utilization away from high-intensity hospital-based services. The QII reflects improvements in clinical outcomes and service quality. The HMI represents the provision of preventive and population-oriented health services.

Examining these indices separately allows the analysis to assess whether smart city development is associated with changes across different dimensions of healthcare system performance.

## Results

### Descriptive statistics

Table 1 presents descriptive statistics for the main variables used in the empirical analysis. The final sample consists of 31 provinces observed from 2013 to 2024, yielding a total of 372 province–year observations.

**TABLE 1:** Descriptive statistics of main variables used in the empirical analysis. Smart City Development and Medical Service Performance Study, China, 2013-2024.

Variable	N	Mean	Std. Dev.	Min	Max
Medical service performance index (MSPI)	372	0.000	0.873	−2.11	2.54
Smart city exposure	372	0.412	0.271	0.000	1.000
GDP *per capita* (RMB)	372	67,834	37,215	14,532	189,332
Urbanization rate	372	0.612	0.124	0.350	0.890
Population density(persons/km^2^)	372	458.7	612.3	8.4	3,847.6
Fiscal expenditure *per capita* (RMB)	372	12,654	5402	4,133	34,222

Smart city exposure measures the proportion of smart city pilot cities within each province.

The Medical Service Performance Index (MSPI) shows substantial variation across provinces and over time. The index ranges from −2.11 to 2.54, with a standard deviation of 0.873. This dispersion suggests considerable heterogeneity in healthcare system performance across Chinese provinces. Such variation reflects differences not only in healthcare utilization patterns but also in service quality and preventive health management coverage.

Smart city exposure also exhibits considerable variation across provinces. The variable ranges from 0 to 1, with an average value of 0.412 and a standard deviation of 0.271. This pattern reflects the gradual expansion of China’s national smart city pilot program since its launch in 2013. In the early years of the program, several provinces recorded zero exposure because no cities had yet been designated as pilot cities. As additional cities were gradually incorporated into the program, the exposure level increased across many provinces, generating both cross-sectional and temporal variation suitable for panel analysis.

The control variables further illustrate the diversity of regional socioeconomic conditions. GDP *per capita* varies widely, ranging from 14,532 RMB to 189,332 RMB. Urbanization rates range from 0.35 to 0.89, reflecting large differences in the spatial distribution of population and infrastructure across provinces. Population density also varies substantially, from sparsely populated western regions to highly concentrated coastal provinces. Fiscal expenditure *per capita* ranges from 4,133 RMB to 34,222 RMB, indicating significant variation in local government fiscal capacity. These disparities underscore the importance of controlling for regional socioeconomic conditions when examining the relationship between smart city development and healthcare system performance.

### Baseline results

Table 2 reports the baseline estimates from the two-way fixed effects models examining the relationship between smart city exposure and medical service performance.

**TABLE 2:** Association between smart city exposure and medical service performance based on two-way fixed effects regression models. Smart City Development and Medical Service Performance Study, China, 2013-2024.

Variables	(1)	(2)	(3)
Smart city exposure	0.182***	0.135**	0.109*
GDP *per capita*	​	0.214***	0.207***
Urbanization	​	0.165**	0.149*
Population density	​	0.031	0.028
Fiscal expenditure	​	0.097*	0.083
Province FE	Yes	Yes	Yes
Year FE	Yes	Yes	Yes
Observations	372	372	372
R^2^	0.41	0.48	0.51

Robust standard errors clustered at the provincial level are reported in parentheses. *, **, and *** denote statistical significance at the 10%, 5%, and 1% levels, respectively.

In the baseline specification (column 1), smart city exposure is positively associated with MSPI, with a coefficient of 0.182, which is statistically significant at the 1% level. This result suggests that provinces with greater exposure to smart city initiatives tend to exhibit higher levels of healthcare system performance.

After introducing socioeconomic control variables (column 2), the estimated coefficient decreases to 0.135, although it remains statistically significant at the 5% level. The reduction in magnitude suggests that part of the baseline association may reflect underlying regional economic and demographic characteristics.

When additional controls are included (column 3), the coefficient further declines to 0.109, remaining statistically significant at the 10% level. Despite the gradual decline in magnitude across specifications, the coefficient remains consistently positive, indicating a stable association between smart city development and healthcare system performance.

The control variables also exhibit patterns broadly consistent with theoretical expectations. GDP *per capita* is positively associated with MSPI, suggesting that economically more developed provinces tend to possess stronger healthcare infrastructure and service capacity. Urbanization rate also shows a positive and statistically significant relationship with MSPI, which may reflect improved access to healthcare services and more efficient allocation of medical resources in more urbanized regions. Fiscal expenditure *per capita* displays a weakly positive relationship with healthcare performance, indicating that stronger fiscal capacity may support public health service provision. In contrast, population density does not exhibit a statistically significant relationship with MSPI in the baseline models.

Overall, the baseline results provide consistent evidence that greater exposure to smart city initiatives is associated with improved healthcare system performance at the provincial level.

### Mechanism analysis

To explore the potential channels through which smart city development may influence healthcare system performance, the analysis further examines three mechanism-based indices: service substitution (SSI), quality improvement (QII), and health management (HMI). These indices correspond to the three conceptual dimensions underlying the Medical Service Performance Index.

Table 3 indicates that smart city exposure is significantly associated with all three mechanism indicators. The coefficient for the service substitution index is −0.120 and statistically significant at the 1% level. This negative association suggests that smart city initiatives may reduce reliance on high-intensity facility-based healthcare services, possibly by facilitating the use of digital health platforms, remote consultations, and other forms of non-hospital-based medical services.

**TABLE 3:** Relationship between smart city exposure and mechanism indicators of healthcare system performance. Smart City Development and Medical Service Performance Study, China, 2013-2024.

Variables	Healthcare service substitution	Care quality improvement	Preventive health management
Smart city exposure	−0.120***	0.135***	0.184***
Controls	Yes	Yes	Yes
Province fixed effects	Yes	Yes	Yes
Year fixed effects	Yes	Yes	Yes
Province-specific trends	No	No	Yes
Observations	372	372	372
R-squared	0.46	0.44	0.52

Service substitution reflects changes in healthcare utilization structure. Quality improvement reflects improvements in clinical outcomes. Health management captures preventive health service capacity. Statistical significance is denoted as follows: *p < 0.10, **p < 0.05, and ***p < 0.01.

In contrast, smart city exposure is positively associated with both the quality improvement index and the health management index. The coefficient for quality improvement is 0.135, while the coefficient for health management is 0.184, both statistically significant at the 1% level. These findings suggest that smart city development may contribute to improvements in clinical service outcomes as well as the expansion of preventive health management services.

Province-specific trends are included in the health management specification to account for potential long-term regional development trajectories that may influence preventive healthcare services. The inclusion of this specification helps ensure that the estimated relationship between smart city exposure and health management capacity is not driven by long-term regional trends unrelated to digital governance initiatives.

Taken together, these results suggest that smart city development may influence healthcare system performance through multiple complementary channels related to healthcare utilization patterns, service quality, and preventive health management.

### Mechanism decomposition

Table 4 further evaluates whether these mechanisms account for the relationship between smart city exposure and overall healthcare system performance. In this specification, the three mechanism indices are introduced simultaneously into the baseline MSPI regression model.

**TABLE 4:** Mediation analysis examining the mechanisms linking smart city exposure and medical service performance. Smart City Development and Medical Service Performance Study, China, 2013–2024.

Variables	Health management index
Smart city exposure	0.071
Service substitution index	0.283***
Quality improvement index	0.314***
Health management index	0.418***
Controls	Yes
Province FE	Yes
Year FE	Yes
Observations	372
R^2^	0.63

Mechanism variables capture three underlying dimensions of healthcare system performance. Statistical significance is denoted as follows: *p < 0.10, **p < 0.05, and ***p < 0.01.

The results show that all three mechanism variables are significantly associated with MSPI. The coefficients for the service substitution index, quality improvement index, and health management index are 0.283, 0.314, and 0.418 respectively, all statistically significant at the 1% level. These findings indicate that improvements in healthcare utilization structure, service quality, and preventive health management are closely related to overall healthcare system performance.

Once these mechanism variables are included in the regression model, the coefficient for smart city exposure decreases substantially and becomes statistically insignificant. This pattern suggests that the association between smart city development and healthcare system performance operates primarily through the mechanisms captured by these indices rather than through a direct effect.

Overall, the results support the view that smart city initiatives influence healthcare systems through structural changes in service delivery and health management rather than through isolated technological interventions.

### Robustness checks

To assess the stability of the baseline findings, Table 5 reports a series of robustness checks using alternative model specifications. Across these models, the estimated coefficient for smart city exposure remains positive and statistically significant.

**TABLE 5:** Robustness checks of the baseline regression results examining the relationship between smart city exposure and medical service performance. Smart City Development and Medical Service Performance Study, China, 2013-2024.

Variables	(1)	(2)	(3)
Smart city exposure	0.167**	0.142**	0.118*
Controls	Yes	Yes	Yes
Province FE	Yes	Yes	Yes
Year FE	Yes	Yes	Yes
Observations	372	372	372
R^2^	0.45	0.47	0.49

Robust standard errors clustered at the provincial level are reported in parentheses. Statistical significance is denoted as follows: *p < 0.10, **p < 0.05, and ***p < 0.01.

The estimated coefficients range from 0.118 to 0.167 across specifications and remain statistically significant at either the 10% or 5% level. Although the magnitude of the coefficients is slightly smaller than in the baseline models, the direction and significance of the relationship remain consistent.

These results indicate that the positive association between smart city exposure and healthcare system performance is robust to alternative model specifications. Taken together with the baseline and mechanism analyses, the evidence suggests that the diffusion of smart city initiatives is systematically associated with improvements in healthcare system performance across Chinese provinces. These results provide further support for the main empirical finding that higher levels of smart city exposure are associated with improved medical service performance.

## Discussion

Recent research has increasingly emphasized the role of digital transformation in shaping healthcare system performance. Existing studies have identified a range of institutional and structural factors associated with healthcare outcomes, including government health expenditure [35], coordination between local governments and healthcare institutions, private-sector participation [36], regional governance arrangements [37], healthcare infrastructure development [38], the allocation of health human resources [39], and patient engagement in healthcare decision-making [40]. While these studies provide important insights, digitalization is often conceptualized primarily at the level of medical technologies or hospital information systems. In comparison, relatively limited attention has been devoted to how broader digital governance transformations at the urban level may influence healthcare systems.

Against this background, this study provides new empirical evidence from China linking smart city development to healthcare system performance. Using province-level panel data from 2013 to 2024 and a two-way fixed effects framework, the results show that provinces with higher levels of smart city exposure tend to achieve better medical service performance. In the baseline models, the estimated coefficient of smart city exposure ranges from 0.109 to 0.182 across specifications, indicating a stable positive association even after controlling for socioeconomic, demographic, and fiscal characteristics. These findings suggest that the implications of digitalization for healthcare systems extend beyond the adoption of isolated medical technologies and may instead emerge through broader governance transformations associated with smart city initiatives.

The mechanism analysis provides further insight into the pathways through which smart city development may influence healthcare system performance. Rather than operating through a single channel, the results indicate that smart city exposure is associated with changes across several functional dimensions of healthcare systems. In particular, the negative coefficient for the service substitution index (−0.120) suggests a gradual shift away from high-intensity, hospital-centered care toward more appropriate patterns of healthcare utilization. At the same time, the positive associations observed for both the quality improvement index (0.135) and the health management index (0.184) indicate improvements in clinical outcomes and the expansion of preventive health services. These findings imply that smart city initiatives may enhance healthcare performance through improvements in information integration, service coordination, and population-oriented health management.

The results also carry several policy implications. The positive association between smart city exposure and healthcare system performance suggests that digital governance initiatives may play an important role in strengthening the functioning of public service systems. In particular, the relatively strong relationship observed for the health management dimension highlights the potential of smart city initiatives to improve preventive healthcare and community-based health management services. These functions are increasingly important in contexts characterized by population aging and a rising burden of chronic diseases. Moreover, the improvements observed in healthcare utilization patterns and service quality suggest that digital governance reforms may contribute to better coordination of healthcare resources and more efficient service delivery, rather than simply expanding the volume of healthcare services.

Despite these contributions, several limitations should be acknowledged. One potential concern relates to policy selection bias in the designation of smart city pilot cities. Cities with stronger fiscal capacity, more advanced digital infrastructure, and higher governance capability may be more likely to be selected as national smart city pilots. Although the empirical models control for a range of socioeconomic characteristics and include both province and year fixed effects, unobserved time-varying factors may still simultaneously influence smart city development and healthcare system performance. Therefore, the estimated relationships in this study should be interpreted primarily as statistical associations rather than strict causal effects.

In addition, the smart city exposure indicator used in this study captures the proportion of pilot cities within a province but does not directly reflect differences in the implementation intensity or governance quality of smart city initiatives across cities. Some pilot cities may invest more resources or pursue deeper digital governance reforms than others. As a result, the indicator should be interpreted mainly as a measure of policy diffusion rather than a precise measure of implementation effectiveness. Future research could build on this study by using more granular data at the city or healthcare facility level to further examine the mechanisms linking digital governance and healthcare system performance. Additional work could also explore longer-term health outcomes and investigate how institutional design and local governance capacity shape the effectiveness of smart city initiatives in different regional contexts.

### Conclusion

This study provides province-level evidence from China that smart city development is positively associated with medical service performance. Provinces with higher exposure to smart city initiatives consistently exhibit better overall performance of medical service systems, as reflected in the composite Medical Service Performance Index.

The results indicate that these improvements are not limited to changes in service volume, but are linked to broader enhancements in service coordination, care quality, and preventive health management. Rather than functioning as isolated technological upgrades, smart city initiatives appear to support medical service systems by improving the integration of information, governance capacity, and population-oriented service delivery.

From a policy perspective, the findings suggest that smart city development may contribute to health system strengthening when digital governance tools are aligned with primary care and preventive services. In contexts characterized by rapid urbanization and uneven healthcare capacity, integrating smart city strategies into health system planning may offer a complementary pathway to improving medical service performance. Future research using more granular data could further clarify how local implementation and institutional design shape these effects.

## Data Availability

The data used to support the findings of this study are available from the corresponding author upon request.
